# An alluring case of an infected hepatic hydatid cyst: A case report

**DOI:** 10.1016/j.ijscr.2025.111339

**Published:** 2025-04-19

**Authors:** Talha Ahmed, Deepika C.A., Rahul R. Bhat, Sumith Marian Colaco, Yogesh Kumar

**Affiliations:** a Surgical Registrar, North West Deanery, England, United Kingdom; b Department of General Surgery, Kasturba Medical College Mangalore, Manipal Academy of Higher Education, Karnataka, Manipal 576 104, India

**Keywords:** Hydatid cyst, Infected cyst, Liver abscess, Echinococcus granulosus, Pericystectomy

## Abstract

**Introduction and importance:**

Hydatid cyst is a tapeworm infection that most commonly involves the liver and lungs. Other organs that have been found to be involved are the brain, heart, and bones. Humans are accidental, intermediate hosts and become infected after ingesting the eggs of the parasite. The rupture of the cyst can lead to infection which can subsequently lead to the development of an abscess causing complications like peritonitis.

**Case presentation:**

Here we present a case of a 48-year-old female who was known case of hypertension and diabetes, on medication presented with complaints of an irreducible mass in the right hypochondriac region for 6 months associated with a dull aching type of pain for 3 days with associated fever. Imaging revealed the presence of an intact cyst that was suspected to be a hydatid cyst. The patient was taken up for a pericystectomy during which the cyst was found to be infected based on purulent contents and culture of the contents.

**Clinical discussion:**

Hydatid cyst consists of an endocyst and a pericyst. Hence, infection of the cyst is a rare phenomenon and is seen in immunocompromised patients. The presence of an intact cyst seen both intraoperatively and on imaging makes this case a rare occurrence.

**Conclusion:**

Infection of a hydatid cyst is a complication, difficult to diagnose on imaging. Early surgical intervention like incision and drainage of the abscess followed by pericystectomy should be performed, following which appropriate antimicrobial therapy should be started.

## Highlights

• Hydatid disease is a parasitic infection caused by Echinococcus granulosus and often affects the liver and lungs.
• Infected cysts may present with fever and right upper abdominal pain despite intact walls on imaging.
• Even with modern imaging, definitive diagnosis may only occur intraoperatively.
• Surgical pericystectomy is preferred for large or complicated hydatid cysts.
• Targeted antibiotics post-surgery improve outcomes in infected hydatid cysts.

## 1 Introduction

Hydatid disease is a tapeworm infection that spreads by ingestion of Echinococcus granulosus eggs. The adult worm lives parasitically in the intestine of the definitive host, the dog, wolf, and other wild carnivores. The eggs passed in the faeces, are ingested by sheep and other herbivores, which are the intermediate hosts. The intestinal juices then free the egg from its cuticle and are carried by the bloodstream to the liver or lung, where it develops into a hydatid cyst. In each mature hydatid cyst, there may be thousands of scolices that, if released from the hydatid cyst, may form a new cyst if it is planted in a suitable environment [1]. Humans are accidental, intermediate hosts and become infected after ingesting the eggs of the parasite. Hydatid cysts most commonly involve the liver and lungs but may also be found in other organs, including the brain, heart, and bones [[2], [3], [4], [5]]. Complications include infection of the cyst which could further lead to pyogenic liver abscess and cause secondary acute peritonitis [6,7]. Here we present one such case of an infected hydatid cyst with an intact cyst wall. The case has been reported in line with the SCARE Criteria [8].

## 2 Case description

A 48-year-old female presented with complaints of a mass in the right hypochondriac region for 6 months. It was insidious in onset and gradually progressive. It was associated with a localized, dull aching type of pain at the site of the mass for 3 days which was insidious in onset with no exaggerating or relieving factors and was non-radiating in nature. The patient also had a history of fever. She had no complaints of vomiting or constipation. The patient was a known case of hypertension and diabetes was on medications for the same. She had no significant family history, past medical or surgical history. The patient gave no history of allergies or any habits.

On inspection, the abdomen appeared distended with no visible mass or pulsations. On palpation, there was no local rise in temperature but was tender in the right hypochondriac region. A mass measuring 4 cm × 4 cm was palpable in the right hypochondriac region, which moved with respiration. It was cystic in consistency with a smooth surface and was non-reducible. On percussion, a dull note was elicited over the mass and there was no evidence of shifting dullness. On auscultation, normal bowel sounds were heard in all quadrants of the abdomen.

### 2.1 Diagnostic assessment

Routine investigations like complete blood count, and liver, renal, pancreatic, and thyroid profiles revealed significantly elevated levels of alkaline phosphatase along with chronic hyponatremia. Other parameters of the routine investigation were within normal limits. Urine microscopy showed evidence of a trace amount of protein.

Ultrasonography of the abdomen revealed an enlarged liver measuring 22 cm with a well-defined lesion with dimensions of 15.1 cm × 20 cm × 19.2 cm arising from the right lobe of the liver. The lesion had minimal vascularity and contained multiple thin septations with evidence of central stellate hyper-echogenicity, suggestive of a hepatic hydatid cyst. Right-sided pleural effusion was also noted with basal segment consolidation.

**Fig. 1:**
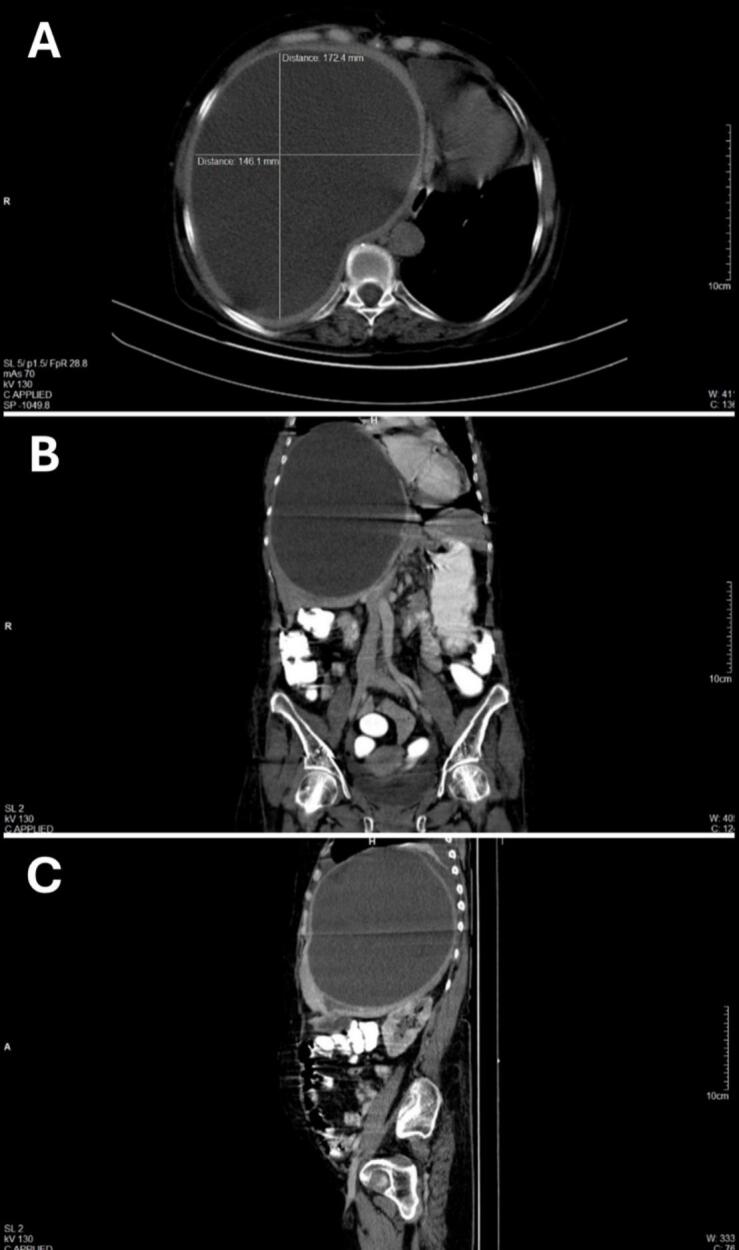
Triple phase CE-CT scan of the abdomen- (A) Axial, (B) coronal and (C) sagittal views showing a hypoechoic cystic lesion involving the right lobe of the liver.

A triple phase contrast-enhanced CT scan of the abdomen showed a hypoechoic cystic lesion involving the right lobe of the liver with hypoechoic contents showing clear fluid and an enhancing hyperechoic cyst wall with no internal debris. There was no evidence of malignancy or involvement of the biliary radicals (Fig. 1).

An upper GI endoscopy was done which was normal, ruling out malignancy.

### 2.2 Therapeutic intervention

Given the hyponatremia, the patient's sodium levels were optimized and was diagnosed as drug-induced hyponatremia due to the diuretics she was taking for her hypertension. The patient was then withdrawn from the diuretics and started on an alternate group of antihypertensives.

**Fig. 2:**
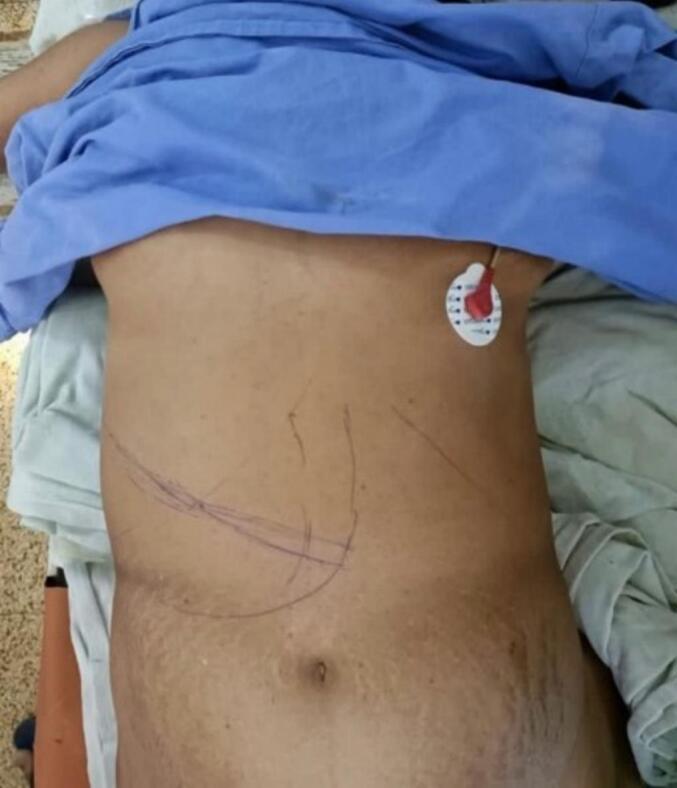
Pre-op image showing marking of mass for pericystectomy.

With the differential of hepatic hydatid cyst in mind, the patient was advised pericystectomy. The complications of the procedure including hemorrhage, hypotension, the chance of liver injury, portal injury, lung pleural injury, pneumothorax, need for thoracotomy, and need for insertion of intercostal drainage catheter insertion were conveyed. Oral 400 mg Albendazole B.D. was administered daily to the patient for about 2 weeks before the procedure. After thorough pre-operative optimization and preparation, the patient was taken up for a pericystectomy of the suspected hydatid cyst (Fig. 2).

**Fig. 3:**
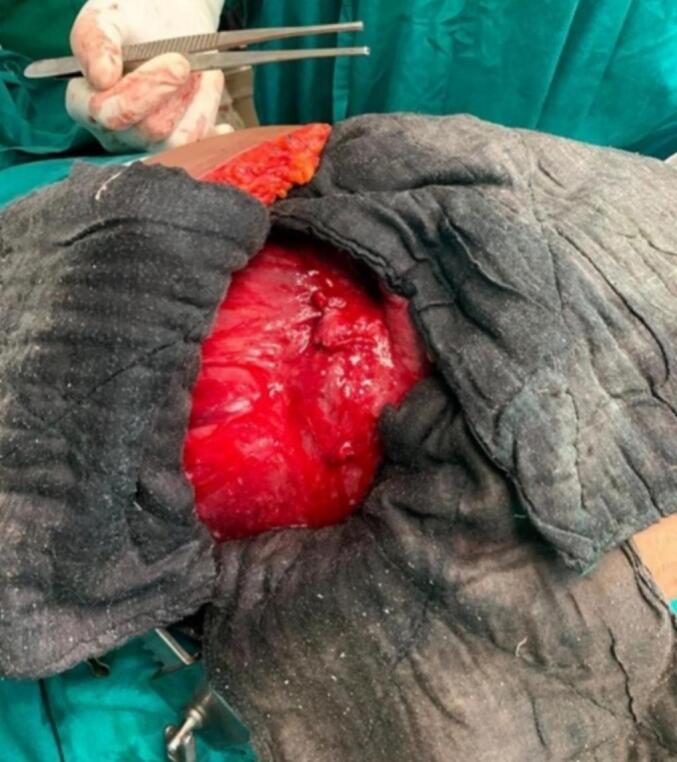
Intraoperative visualization of the cyst found on opening the abdomen.

**Fig. 4:**
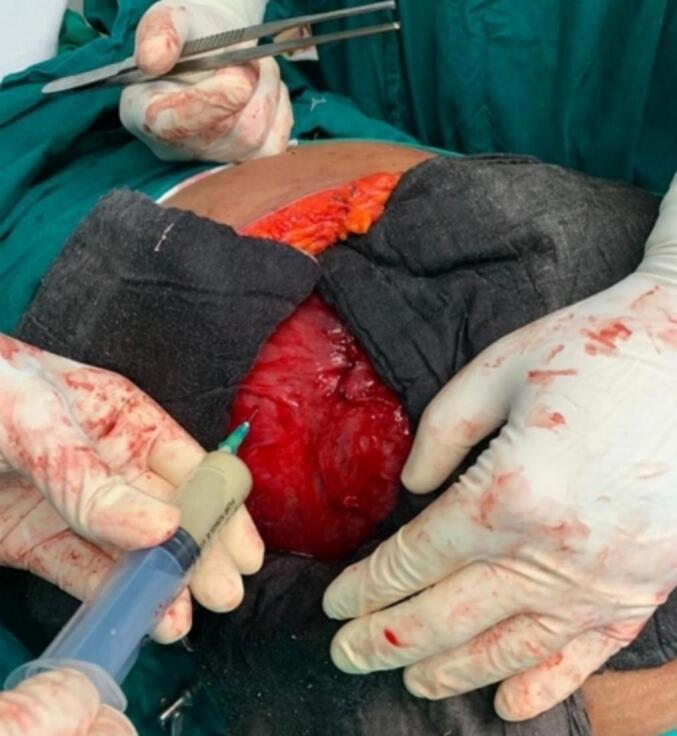
Aspiration of the cyst which revealed the presence of purulent contents.

**Fig. 5:**
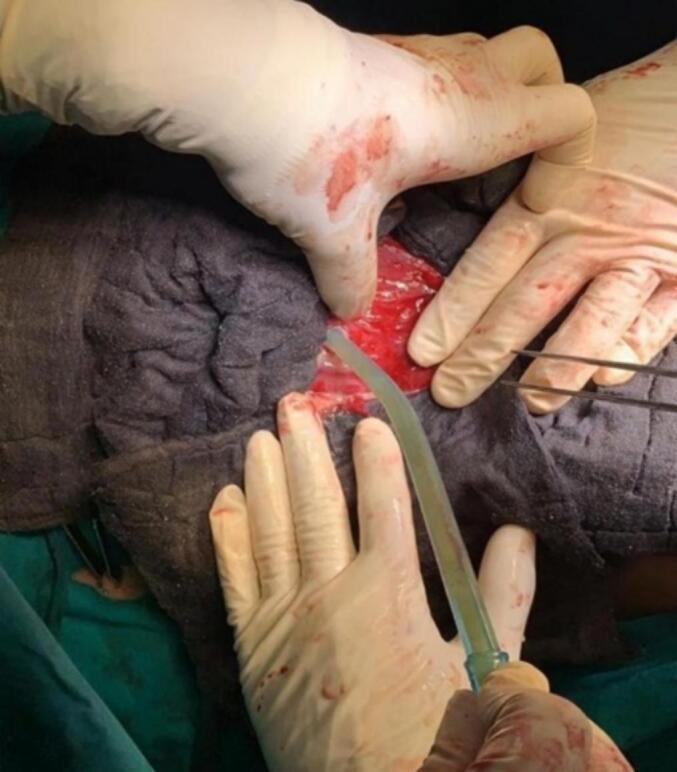
Subsequent drainage of the contents of the cyst following aspiration.

A right sub-costal incision was placed and the abdomen was opened in layers. The mobilized liver capsule was then incised and about 1.5 l of pus was noted which was drained and sent for culture and sensitivity (Fig. 3, Fig. 4, Fig. 5). A Foley's 16-Fr drain catheter was placed in the cyst wall along with a corrugated drain placed at the cystostomy site. A right-sided thoracotomy was performed and an Intercostal Drain (ICD) tube was placed. The diaphragm and the abdomen were closed in layers.

### 2.3 Pathology

On gross examination, the specimen contained multiple pale white mucous membranous tissue bits largest measuring 6 cm × 1.5 cm × 1 cm and the smallest measuring 0.5 cm in diameter.

Histopathology revealed a cyst wall with an outer acellular laminated membrane with calcified protoscolices attached to the membrane and budding from it. Occasional free hooklets were seen. Extensive degenerative changes and calcification were noted. These features were consistent with the diagnosis of a hydatid cyst.

### 2.4 Follow-up and outcomes

Postoperatively, the pus collected intraoperatively was cultured and the growth in the culture was detected as *Escherichia coli*, which was resistant to aztreonam, ampicillin, cefotaxime, ceftazidime, cefuroxime, ciprofloxacin, ofloxacin, and piperacillin. The bacilli were sensitive to amikacin, cefoperazone, chloramphenicol, cotrimoxazole, gentamicin, ertapenem, imipenem, meropenem, and tigecycline. There was no evidence of any post-operative hemorrhage, pneumothorax, or clinical features of hypotension. The operated site healed well with no discharge.

The drain output was regularly monitored and removed on POD 8. The ICD fluid drawn showed the presence of Acinetobacter, which was sensitive to ceftazidime, imipenem, meropenem, and piperacillin. The patient was diagnosed with liver abscess secondary to infection of a hydatid cyst. The choice of antimicrobials administered to the patient were intravenous 1.5 g cefoperazone-sulbactam B.D., 100 ml (500 mg) metronidazole T.I.D., and 40 mg pantoprazole B.D. for seven days.

Ultrasound of the abdomen done on POD 6 revealed hepatomegaly with a multilocular collection of dimensions 8 cm × 6 cm in the right lobe of the liver extending up to the subdiaphragmatic area suggestive of residual cyst collection. Bilateral pleural effusion was also noted. Another scan done on POD 9 revealed similar liver findings with bilateral non-tappable pleural effusion.

The patient was advised 400 mg albendazole B.D. and 200 mg cefixime B.D. oral tablets for 2 weeks on discharge.

## 3 Discussion

Hydatid cyst is a common worldwide infection that leads to the formation of cysts, predominantly located in the liver. About one-fourth of the articles published in the past 60 years were found to be published in the past decade. This increase in the number of publications could be attributed to the increase in diagnostic suspicion and better access to diagnostic technology. Anaphylaxis and infection of the cyst are the common complications that can occur as a result of rupture of the cyst [9,10].

Since the cyst contains a layer of endocyst surrounded by a layer of pericyst, an intact cyst generally remains sterile. The rupture of both of these walls leads to the easy passage of bacteria from the source of infection into the cyst, causing infection within the cyst [11]. Patients with immunocompromised status are more prone to develop liver abscesses following rupture of the hydatid cyst [12]. An infected hydatid cyst usually presents with symptoms of fever and localized pain in the right hypochondrium. The common risk factors for infection in patients with a hydatid cyst include diabetes, steroid use, and liver cirrhosis [13].

Among the available imaging modalities, ultrasonography (USG) of the abdomen is an essential basic radiological investigation, used to detect the presence of a cyst with septations. Furthermore, to delineate the extent of the lesion and demonstrate specific details such as characteristics of the cyst wall and further information on the contents, the use of Computed Tomography (CT) is recommended [11]. However, in our case, despite the use of both USG and CT, there was a diagnostic difficulty because imaging failed to identify the presence of an abscess within the cyst.

The preferred mode of treatment in cases of a hydatid cyst is surgical intervention which is considered comparatively better than the medical line of management. There is no single surgical intervention that is the gold standard for treatment and can vary based on the location and nature of the cyst, including options such as pericystectomy, hepatic resection, marsupialization or mere conservative drainage for these cysts [[14], [15], [16], [17]]. The use of puncture, aspiration, injection, and re-aspiration (PAIR) can also be done in these cysts, however, they are known to more commonly develop both recurrence and complications compared to other surgical interventions [18]. The medical management includes the use of albendazole, which has been shown to play a significant role in preventing recurrence, reducing the size, and even resulting in the death of these cysts [19]. Cultures obtained from these cysts usually show the presence of *Escherichia coli*. Postoperative management by administration of antibiotics based on antimicrobial sensitivity has been shown to improve the outcomes in these patients [9].

## 4 Conclusion

In conclusion, infection of a hepatic hydatid cyst is possible and could be diagnostically difficult to identify as seen in our case. Hence, the possibility of an infected cyst should be kept in mind despite the absence of clinical suspicion with findings of an intact cyst on imaging. Early surgical intervention like incision and drainage of the abscess followed by pericystectomy should be performed, following which appropriate antimicrobial therapy should be started based on the findings of antimicrobial susceptibility testing.

## Author contribution

Dr. Talha Ahmed– Study concept, Study design, Data collection, Writing of original draft, Surgical Operation- Study concept, writing, and editing of article.

Dr. Deepika C A- Study concept, writing, and editing of article.

Dr. Rahul R Bhat- Study concept, writing, and editing of article.

Dr. Sumith Marian Colaco– Study concept, writing, and editing of article.

Dr. Yogesh Kumar- Study concept, writing, and editing of article.

## Informed consent

Written informed consent was obtained from the patient for publication of this case report and accompanying images. A copy of the written consent is available for review by the Editor-in-Chief of this journal on request.

## Ethical approval

Ethical approval was received from the Institutional Ethics Committee of Kasturba Medical College, Mangalore (Reg. no. ECR/541/Inst/ KA/2014/RR-17).

## Guarantor

Dr. Talha Ahmed.

## Research registration number

N/A.

## Funding

No sources of funding.

## Conflict of interest statement

The authors have NO affiliations with or involvement in any organization or entity with any financial interest (such as honoraria; educational grants; participation in speakers' bureaus; membership, employment, consultancies, stock ownership, or other equity interest; and expert testimony or patent-licensing arrangements), or nonfinancial interest (such as personal or professional relationships, affiliations, knowledge or beliefs) in the subject matter or materials discussed in this manuscript.
